# Detection of Parameter Change in Random Coefficient Integer-Valued Autoregressive Models

**DOI:** 10.3390/e20020107

**Published:** 2018-02-06

**Authors:** Jiwon Kang

**Affiliations:** Research Institute for Basic Sciences, Jeju National University, Jeju 63243, Korea; jwkang@jejunu.ac.kr; Tel.: +82-64-754-3596

**Keywords:** integer-valued time series, RCINAR models, test for parameter change, conditional least squares estimator

## Abstract

This paper considers the problem of testing for parameter change in random coefficient integer-valued autoregressive models. To overcome some size distortions of the existing estimate-based cumulative sum (CUSUM) test, we suggest estimating function-based test and residual-based CUSUM test. More specifically, we employ the estimating function of the conditional least squares estimator. Under the regularity conditions and the null hypothesis, we derive their limiting distributions, respectively. Simulation results demonstrate the validity of the proposed tests. A real data analysis is performed on the polio incidence data.

## 1. Introduction

In recent years, time series of counts are widely observed in real-world applications, for instance, the monthly number of people with a certain disease, the number of transactions per minute of some stock, the number of accidents per a day and so on. Among the existing models for analyzing those data sets, autoregressive moving average (ARMA)-type models based on a thinning operator, referred to as integer-valued ARMA models, are still popular since ARMA-type models provide a convenient way to transfer the classical ARMA recursion to discrete-valued time series (cf. Fokianos [1]). Reviews for these models are given by McKenzie [2], Weiß [3], Scotto et al. [4] and references cited therein.

As is addressed in Kang and Lee [5], integer-valued time series, particularly in epidemiology, often undergo a significant change as a result of changes in the quality of health care and the state of patients’ health. It is well known that such a change can affect the statistical inference undesirably and ignoring a parameter change can lead to a false conclusion. Thus, the change point detection has attracted a lot of attention. In the field of integer-valued time series, Fokianos and Fried [6,7] investigated a testing procedure for the detection of intervention effects in linear and log-linear Poisson autoregressive (AR) models. Szabó [8] proposed the test for a change in several crucial parameters of integer-valued autoregressive (INAR) (p) models. Kang and Lee [5,9] constructed the estimate-based cumulative sum (CUSUM) tests for parameter change in random coefficient integer-valued autoregressive (RCINAR) models and Poisson AR models, respectively. More recently, Pap and Szabó [10] developed change detection methods for INAR(p) processes in general and provided the results available under the alternative hypothesis. Doukhan and Kengne [11] proposed two tests based on the likelihood of the observations in a general class of Poisson AR models. Hudecová et al. [12,13,14,15] studied methods for detecting structural changes in INAR and Poisson AR models incorporating the empirical probability generating function and Kang and Song [16] constructed the score test in Poisson AR models.

This study is concerned with change point problem in RCINAR models. The random coefficient setting reflects that the autoregressive coefficient may vary randomly over time due to environmental factors (cf. Zheng et al. [17], Leonenko et al. [18] and Gomes and Canto e Castro [19]). As aforementioned, Kang and Lee [9] developed the estimate-based CUSUM test in RCINAR models. Here, they constructed the test statistics based on the differences ${\hat{\theta }}_{k}-{\hat{\theta }}_{n}$ to detect a change in parameter θ, where ${\hat{\theta }}_{k}$ denotes the estimator based on $\left\{X_{1},\cdots ,X_{k}\right\}$. Their test statistic is very intuitive, but it has a drawback in that it produces severe size distortions especially when true parameter lies near the boundary of parameter space. This motivates us to consider alternative methods. In this paper, we propose an estimating function (EF)-based test and residual-based CUSUM test.

The EF-based test is constructed using the partial sum of estimating function and may be referred to as Z-process method as in Negri and Nishiyama [20]. Score test for parameter change is an example of the EF-based test. Indeed, the score test has been studied by several authors. See, for example, Horváth and Parzen [21], Berkes et al. [22], and Song and Kang [23]. Residual-based CUSUM test has been used popularly due to its ease of implementation. Because the residuals can avoid dependence structure in time series observations, it usually produces stable sizes. See, for example, Lee et al. [24], Kulperger and Yu [25], Kang and Lee [5] and so on. In this study, we use the conditional least squares estimator (CLSE) to estimate the RCINAR models. Hence, our EF-based test is proposed using the EF of the CLSE. For residual-based test, we define residual of RCINAR model as the difference between the observation and its conditional expectation, and then construct CUSUM test.

This paper is organized as follows. In Section 2, we review the CLSE for RCINAR models and its asymptotic properties. In Section 3, we present the EF-based test and residual-based CUSUM test and derive their limiting null distributions. In Section 4, we perform a simulation study to see the finite sample performance. In Section 5, we apply our tests to the polio incidence data for illustration. Section 6 concludes the paper. All the proofs for the results in Section 3 are provided in the Appendix.

## 2. CLSE for RCINAR Models

First, the thinning operator is defined as follows: Let *X* be an integer-valued random variable and ϕ∈[0,1], then the thinning operator “∘” takes the form $\phi \circ X=\sum_{i=1}^{X}B_{i}$ where $\left\{B_{i}\right\}$ is an i.i.d. Bernoulli random sequence with mean ϕ that is independent of *X* (cf. Steutal and Van Harn [26]). With this operator, the RCINAR model is defined by

(1) $$\begin{array}{c} X_{t}=\phi_{t}\circ X_{t-1}+Z_{t},\;\;t\geq 1, \end{array}$$

where $\left\{\phi_{t}\right\}$ is an i.i.d. sequence with range [0, 1), $\left\{Z_{t}\right\}$ is an i.i.d. sequence with range $N_{0}=N\cup \left\{0\right\}$ that is independent of $\left\{\phi_{t}\right\}$ and the counting sequences $\left\{B_{i}\right\}$ involved in $\phi_{t}\circ X_{t-1}$ for t≥1 are mutually independent and independent of $\left\{Z_{t}\right\}$. Note that, conditioned on $X_{t-1}$ and $\phi_{t}$, $\phi_{t}\circ X_{t-1}$ follows a binomial distribution with parameters $X_{t-1}$ and $\phi_{t}$. Assume that $E(\phi_{t}^{2})<\infty$ and $E(Z_{t}^{4})<\infty$. According to Proposition 2.2 of Zheng et al. [17], under the assumptions, the Markov chain $\left\{X_{t}\right\}$ has a unique stationary distribution. From now on, we suppose that the distribution of the initial value $X_{0}$ coincides with this uniquely existing stationary distribution, yielding that the sequence $\left\{X_{t}\right\}$ is strictly stationary.

Let $\theta ={\left(\phi ,\lambda \right)}^{T}={\left(E\left(\phi_{t}\right),E\left(Z_{t}\right)\right)}^{T}$, and denote the true value of θ by $\theta_{0}={\left(\phi_{0},\lambda_{0}\right)}^{T}$. To estimate the unknown parameters, we consider the CLSE. Suppose that $X_{0},X_{1},\ldots ,X_{n}$ from the model (1) are observed. Then, the CLSE ${\hat{\theta }}_{n}$ is obtained by minimizing the conditional sum of squares

$$\begin{array}{c} S_{n}\left(\theta \right)=\sum_{t=1}^{n}{\left(X_{t}-E\left(X_{t}|X_{t-1}\right)\right)}^{2}=\sum_{t=1}^{n}{\left(X_{t}-\phi X_{t-1}-\lambda \right)}^{2}:=\sum_{t=1}^{n}\epsilon_{t}^{2}\left(\theta \right) \end{array}$$

over $R^{2}$, and is given by

$$\begin{array}{ccc} {\hat{\phi }}_{n} & = & \frac{n\sum_{t=1}^{n}X_{t}X_{t-1}-\left(\sum_{t=1}^{n}X_{t-1}\right)\left(\sum_{t=1}^{n}X_{t}\right)}{n\sum_{t=1}^{n}X_{t-1}^{2}-{\left(\sum_{t=1}^{n}X_{t-1}\right)}^{2}} \end{array}$$

and

$$\begin{array}{ccc} {\hat{\lambda }}_{n} & = & \frac{1}{n}\left(\sum_{t=1}^{n}X_{t}-{\hat{\phi }}_{n}\sum_{t=1}^{n}X_{t-1}\right). \end{array}$$

Throughout the paper, we use $\partial_{\theta }$ and $\partial_{\theta }^{2}$ to denote ∂/∂θ and $\partial^{2}/\partial \theta \partial \theta^{T}$, respectively. The symbol ||·|| denotes the $l_{2}$ norm for matrices and vectors and E(·) is taken under $\theta_{0}$. The symbols $\overset{d}{\to }$ and $\overset{p}{\to }$ denote convergence in distribution and convergence in probability, respectively. The almost sure convergence is written as “a.s.”.

We define the function g(·,·) by g(θ,x)=ϕx+λ, then $S_{n}\left(\theta \right)$ can be written in the form $\sum_{t=1}^{n}{\left(X_{t}-g\left(\theta ,X_{t-1}\right)\right)}^{2}$. And then, the following result can be established by checking the regularity conditions in Klimko and Nelson [27].

**Theorem** **1.** *We have that ${\hat{\theta }}_{n}$ converges to $\theta_{0}$ almost surely and*

$$\begin{array}{c} \sqrt{n}\left({\hat{\theta }}_{n}-\theta_{0}\right)\overset{d}{⟶}N\left(0,V^{-1}WV^{-1}\right) \end{array}$$

*where V and W are positive definite matrices defined by*

$$\begin{array}{ccc} V: & = & E\left(\partial_{\theta }g\left(\theta_{0},X_{0}\right)\partial_{\theta^{T}}g\left(\theta_{0},X_{0}\right)\right)=\left(\begin{array}{cc} E(X_{0}^{2}) & E(X_{0})\\ E(X_{0}) & 1 \end{array}\right)\\ W: & = & E\left(u_{1}^{2}\left(\theta_{0}\right)\partial_{\theta }g\left(\theta_{0},X_{0}\right)\partial_{\theta^{T}}g\left(\theta_{0},X_{0}\right)\right)=\left(\begin{array}{cc} E(X_{0}^{2}{\left(X_{1}-\phi_{0}X_{0}-\lambda_{0}\right)}^{2}) & E(X_{0}{\left(X_{1}-\phi_{0}X_{0}-\lambda_{0}\right)}^{2})\\ E(X_{0}{\left(X_{1}-\phi_{0}X_{0}-\lambda_{0}\right)}^{2}) & E({\left(X_{1}-\phi_{0}X_{0}-\lambda_{0}\right)}^{2}) \end{array}\right) \end{array}$$

*with* $u_{1}\left(\theta_{0}\right)=X_{1}-E\left(X_{1}|X_{0}\right)=X_{1}-\phi_{0}X_{0}-\lambda_{0}$.

## 3. Parameter Change Test for RCINAR Models

In this section, we consider the problem of testing the following hypotheses:

$$\begin{array}{c} H_{0}:\theta \mathrm{does}\mathrm{not}\mathrm{change}\mathrm{over}\;X_{1},\cdots ,X_{n}\;vs.\\ H_{1}:\mathrm{there}\mathrm{exists}\mathrm{an}\mathrm{integer}\;k^{*}\in \left\{1,\cdots ,n-1\right\}\mathrm{such}\mathrm{that}\;\\ \;\;\;\;\;\;\;\theta \mathrm{does}\mathrm{not}\mathrm{change}\mathrm{over}\;X_{1},\cdots ,X_{k^{*}}\;\mathrm{and}\mathrm{over}\;X_{k^{*}+1},\cdots ,X_{n}\;\mathrm{but}\mathrm{changes}\mathrm{over}\;X_{1},\cdots ,X_{n},\\ \;\;\;\;\;\;\;\mathrm{and}\;Z_{1},\cdots ,Z_{k^{*}}\mathrm{are}\mathrm{identically}\mathrm{distributed}\mathrm{and}\;Z_{k^{*}+1},\cdots ,Z_{n}\mathrm{are}\mathrm{identically}\mathrm{distributed}. \end{array}$$

To this end, we employ the EF-based test and residual-based CUSUM test.

### 3.1. EF-Based Test

First, we consider the EF-based test using the partial sum process of the following estimating function:

$$\begin{array}{c} \partial_{\theta }S_{n}\left(\theta \right)=\sum_{t=1}^{n}\partial_{\theta }\epsilon_{t}^{2}\left(\theta \right)=\sum_{t=1}^{n}2\left(X_{t}-\phi X_{t-1}-\lambda \right)\left(\begin{array}{c} -X_{t-1}\\ -1 \end{array}\right). \end{array}$$

As the estimate-based test in Kang and Lee [9] is constructed based on the differences ${\hat{\theta }}_{k}-{\hat{\theta }}_{n}$, we construct a test statistic using the differences $\partial_{\theta }S_{k}\left({\hat{\theta }}_{n}\right)-\partial_{\theta }S_{n}\left({\hat{\theta }}_{n}\right)$. Noting the fact that $\partial_{\theta }S_{n}\left({\hat{\theta }}_{n}\right)=0$, we can see that the differences become $\partial_{\theta }S_{k}\left({\hat{\theta }}_{n}\right)$. Then, the test statistic is proposed as the maximum value of a function of $\partial_{\theta }S_{k}\left({\hat{\theta }}_{n}\right)$. To derive its limiting distribution, it is needed to obtain the limiting distribution of $\partial_{\theta }S_{\left[ns\right]}\left({\hat{\theta }}_{n}\right)$ for each s∈[0,1].

By Taylor’s theorem, we have that for each s∈[0,1],

$$\frac{1}{\sqrt{n}}\partial_{\theta }S_{\left[ns\right]}\left({\hat{\theta }}_{n}\right)=\frac{1}{\sqrt{n}}\partial_{\theta }S_{\left[ns\right]}\left(\theta_{0}\right)+\frac{1}{n}\partial_{\theta }^{2}S_{\left[ns\right]}\left(\theta_{n,s}^{*}\right)\sqrt{n}\left({\hat{\theta }}_{n}-\theta_{0}\right),$$

where $\theta_{n,s}^{*}$ is an intermediate point between $\theta_{0}$ and ${\hat{\theta }}_{n}$ and [ns] is the integer part of ns. Here, noting the fact that $\partial_{\theta }^{2}S_{\left[ns\right]}\left(\theta \right)$ does not depend on the parameter θ, we have $\partial_{\theta }^{2}S_{\left[ns\right]}\left(\theta_{n,s}^{*}\right)=\partial_{\theta }^{2}S_{\left[ns\right]}\left(\theta_{0}\right)$ for all s∈[0,1]. Thus, we can see that for each s∈[0,1],

(2) $$\begin{array}{c} \frac{1}{\sqrt{n}}\partial_{\theta }S_{\left[ns\right]}\left({\hat{\theta }}_{n}\right)=\frac{1}{\sqrt{n}}\partial_{\theta }S_{\left[ns\right]}\left(\theta_{0}\right)+\frac{1}{n}\partial_{\theta }^{2}S_{\left[ns\right]}\left(\theta_{0}\right)\sqrt{n}\left({\hat{\theta }}_{n}-\theta_{0}\right). \end{array}$$

It follows from (2) and $\partial_{\theta }S_{n}\left({\hat{\theta }}_{n}\right)=0$ that for s=1,

$$\begin{array}{c} 0=\frac{1}{\sqrt{n}}\;\partial_{\theta }S_{n}\left({\hat{\theta }}_{n}\right)=\frac{1}{\sqrt{n}}\;\partial_{\theta }S_{n}\left(\theta_{0}\right)+\frac{1}{n}\partial_{\theta }^{2}S_{n}\left(\theta_{0}\right)\sqrt{n}\left({\hat{\theta }}_{n}-\theta_{0}\right). \end{array}$$

Let $V_{n}:=\partial_{\theta }^{2}S_{n}\left(\theta_{0}\right)/n$, then $\frac{1}{2}V_{n}\to V$ a.s. by the ergodicity of $X_{t}$. The above equation can be rewritten as

(3) $$\begin{array}{c} \sqrt{n}\left({\hat{\theta }}_{n}-\theta_{0}\right)=-V^{-1}\frac{1}{2\sqrt{n}}\;\partial_{\theta }S_{n}\left(\theta_{0}\right)-V^{-1}\left(\frac{1}{2}V_{n}-V\right)\sqrt{n}\left({\hat{\theta }}_{n}-\theta_{0}\right). \end{array}$$

Consequently, from (2) and (3), we can write that

$$\begin{array}{c} \frac{1}{2\sqrt{n}}\partial_{\theta }S_{\left[ns\right]}\left({\hat{\theta }}_{n}\right)=\frac{1}{2\sqrt{n}}\partial_{\theta }S_{\left[ns\right]}\left(\theta_{0}\right)-\frac{\left[ns\right]}{n}\frac{1}{2\sqrt{n}}\;\partial_{\theta }S_{n}\left(\theta_{0}\right)+I_{n}+II_{n} \end{array}$$

where

$$\begin{array}{ccc} I_{n} & := & \frac{\left[ns\right]}{n}\frac{1}{2\sqrt{n}}\;\partial_{\theta }S_{n}\left(\theta_{0}\right)-\frac{1}{2n}\partial_{\theta }^{2}S_{\left[ns\right]}\left(\theta_{0}\right)V^{-1}\;\frac{1}{2\sqrt{n}}\;\partial_{\theta }S_{n}\left(\theta_{0}\right),\\ II_{n} & := & \frac{1}{2n}\partial_{\theta }^{2}S_{\left[ns\right]}\left(\theta_{0}\right)V^{-1}\left(V-\frac{1}{2}V_{n}\right)\sqrt{n}\left({\hat{\theta }}_{n}-\theta_{0}\right). \end{array}$$

In fact, it can be verified that

$$W^{-\frac{1}{2}}\left(\frac{1}{2\sqrt{n}}\partial_{\theta }S_{\left[ns\right]}\left(\theta_{0}\right)-\frac{\left[ns\right]}{n}\frac{1}{2\sqrt{n}}\;\partial_{\theta }S_{n}\left(\theta_{0}\right)\right)\overset{w}{⟶}B_{2}^{\circ }\left(s\right)\;\mathrm{in}\;\;D\;\left(\left[0,1\right],\;R^{2}\right)$$

where $B_{2}^{o}$ is a 2-dimensional standard Brownian bridge (see Lemma A1 in the Appendix). Here, D be the function space with respect to the *Skorohod* topology and the symbol $\overset{w}{\to }$ denotes the weak convergence in function space. Here, $W^{-\frac{1}{2}}$ denotes the inverse of the unique positive definite square root of the positive definite matrix *W*. Furthermore, $I_{n}$ and $II_{n}$ are asymptotically negligible (see Lemmas A2 and A3 in the Appendix, respectively). Hence, combining the above arguments, we obtain our first main result.

**Theorem** **2.** *Under* $H_{0}$, *we have*

$$\begin{array}{c} W^{-\frac{1}{2}}\frac{1}{2\sqrt{n}}\partial_{\theta }S_{\left[ns\right]}\left({\hat{\theta }}_{n}\right)\overset{w}{⟶}\;B_{2}^{o}\left(s\right)\;\mathrm{in}\;\;D\;\left(\left[0,1\right],\;R^{2}\right)\;, \end{array}$$

*thus*

$$\begin{array}{c} T_{n}^{EF}:=\max_{1\leq k\leq n}T_{n,k}^{EF}=\max_{1\leq k\leq n}\frac{1}{4n}\partial_{\theta }S_{k}{\left({\hat{\theta }}_{n}\right)}^{T}{\hat{W}}_{n}^{-1}\partial_{\theta }S_{k}\left({\hat{\theta }}_{n}\right)\overset{d}{⟶}\underset{0\leq s\leq 1}{\sup}∥B_{2}^{o}\left(s\right){∥}_{2}^{2}, \end{array}$$

*where* ${\hat{W}}_{n}$ *is a consistent estimator of W. We reject* $H_{0}$ *if* $T_{n}^{EF}$ *is large*.

**Remark** **1.** *As a consistent estimator of W, one can consider to use*

$$\begin{array}{c} {\hat{W}}_{n}=\frac{1}{n}\left(\begin{array}{cc} \sum_{t=1}^{n}X_{t-1}^{2}{\left(X_{t}-{\hat{\phi }}_{n}X_{t-1}-{\hat{\lambda }}_{n}\right)}^{2} & \sum_{t=1}^{n}X_{t-1}{\left(X_{t}-{\hat{\phi }}_{n}X_{t-1}-{\hat{\lambda }}_{n}\right)}^{2}\\ \sum_{t=1}^{n}X_{t-1}{\left(X_{t}-{\hat{\phi }}_{n}X_{t-1}-{\hat{\lambda }}_{n}\right)}^{2} & \sum_{t=1}^{n}{\left(X_{t}-{\hat{\phi }}_{n}X_{t-1}-{\hat{\lambda }}_{n}\right)}^{2} \end{array}\right). \end{array}$$

### 3.2. Residual-Based CUSUM Test

Instead of the EF-based test, we consider the test statistic based on the residuals, which may be defined as the difference between $X_{t}$ and its conditional expectation (cf. Freeland and McCabe [28]). For RCINAR models, the residuals are obtained as $\epsilon_{t}\left(\theta_{0}\right)=X_{t}-\phi_{0}X_{t-1}-\lambda_{0}$. Let $F_{t}$ be the σ-field generated by $\left\{X_{s};s\leq t\right\}$. Since $\left\{\epsilon_{t}\left(\theta_{0}\right),F_{t},1\leq t\leq n\right\}$ forms a sequence of martingale differences, the invariance principle shows that

(4) $$\begin{array}{c} \max_{1\leq k\leq n}\frac{1}{\sqrt{n}\tau }\left|\sum_{t=1}^{k}\epsilon_{t}\left(\theta_{0}\right)-\frac{k}{n}\sum_{t=1}^{n}\epsilon_{t}\left(\theta_{0}\right)\right|\overset{d}{⟶}\underset{0\leq s\leq 1}{\sup}\left|B_{1}^{\circ }\left(s\right)\right|, \end{array}$$

where $\tau^{2}=Var\left(\epsilon_{1}\left(\theta_{0}\right)\right)$. This allows us to construct the residual-based CUSUM test. Here, we replace the residuals with $\epsilon_{t}\left({\hat{\theta }}_{n}\right)=X_{t}-{\hat{\phi }}_{n}X_{t-1}-{\hat{\lambda }}_{n}$, where ${\hat{\phi }}_{n}$ and ${\hat{\lambda }}_{n}$ are the CLSE of $\phi_{0}$ and $\lambda_{0}$, respectively. Using the fact that $\sum_{t=1}^{n}\epsilon_{t}\left({\hat{\theta }}_{n}\right)=0$, we propose the test statistic as follows:

$$\max_{1\leq k\leq n}\frac{1}{\sqrt{n}\tau }\left|\sum_{t=1}^{k}\epsilon_{t}\left({\hat{\theta }}_{n}\right)\right|.$$

From Lemmas A4 and A5, we can see that

$$\begin{array}{c} \max_{1\leq k\leq n}\frac{1}{\sqrt{n}}\left|\sum_{t=1}^{k}\left(\epsilon_{t}\left({\hat{\theta }}_{n}\right)-\epsilon_{t}\left(\theta_{0}\right)\right)-\frac{k}{n}\sum_{t=1}^{n}\left(\epsilon_{t}\left({\hat{\theta }}_{n}\right)-\epsilon_{t}\left(\theta_{0}\right)\right)\right|=o_{P}\left(1\right) \end{array}$$

and

$$\begin{array}{c} {\hat{\tau }}_{n}^{2}:=\frac{1}{n}\sum_{t=1}^{n}\epsilon_{t}^{2}\left({\hat{\theta }}_{n}\right)\overset{p}{⟶}\tau^{2}, \end{array}$$

respectively. Owing to these and (4), we have the second main result.

**Theorem** **3.** *Under* $H_{0}$, *we have*

$$\begin{array}{c} T_{n}^{R}:=\max_{1\leq k\leq n}T_{n,k}^{R}=\max_{1\leq k\leq n}\frac{1}{\sqrt{n}{\hat{\tau }}_{n}}\left|\sum_{t=1}^{k}\epsilon_{t}\left({\hat{\theta }}_{n}\right)\right|\overset{d}{⟶}\underset{0\leq s\leq 1}{\sup}\left|B_{1}^{\circ }\left(s\right)\right|. \end{array}$$

*We reject* $H_{0}$ *if* $T_{n}^{R}$ *is large*.

## 4. Simulation Results

In this section, we evaluate the performance of our tests $T_{n}^{EF}$ and $T_{n}^{R}$. For the comparison purpose, we additionally perform the estimate-based CUSUM test, $T_{n}^{CLS}$, of Kang and Lee [9] given by

$$\begin{array}{c} T_{n}^{CLS}:=\max_{1\leq k\leq n}\frac{k^{2}}{n}{\left({\hat{\theta }}_{k}-{\hat{\theta }}_{n}\right)}^{T}{\hat{V}}_{n}{\hat{W}}_{n}^{-1}{\hat{V}}_{n}\left({\hat{\theta }}_{k}-{\hat{\theta }}_{n}\right) \end{array}$$

where

$${\hat{V}}_{n}=\frac{1}{n}\left(\begin{array}{cc} \sum_{t=1}^{n}X_{t-1}^{2} & \sum_{t=1}^{n}X_{t-1}\\ \sum_{t=1}^{n}X_{t-1} & n \end{array}\right).$$

Kang and Lee [9] showed that under $H_{0}$,

$$\begin{array}{c} T_{n}^{CLS}\overset{d}{⟶}\underset{0\leq s\leq 1}{\sup}∥B_{2}^{o}\left(s\right){∥}_{2}^{2}. \end{array}$$

We consider the RCINAR model

(5) $$\begin{array}{c} X_{t}=\phi_{t}\circ X_{t-1}+Z_{t}, \end{array}$$

where $\left\{\phi_{t}\right\}$ is an i.i.d. sequence of Beta random variables with parameters (a,b) and $\left\{Z_{t}\right\}$ is an i.i.d. Poisson sequence with mean λ. Here, we evaluate $T_{n}^{EF}$, $T_{n}^{R}$ and $T_{n}^{CLS}$ with sample sizes n=300,500,1000 at the nominal level 0.05: the associated critical values, obtained through Monte Carlo simulations, are 2.408, 1.353 and 2.408, respectively. For each simulation, the first 1000 initial observations are discarded to avoid initialization effects. The empirical sizes and powers are calculated as the proportion of the number of rejections of the null hypothesis based on 1000 repetitions.

**Figure 1:**
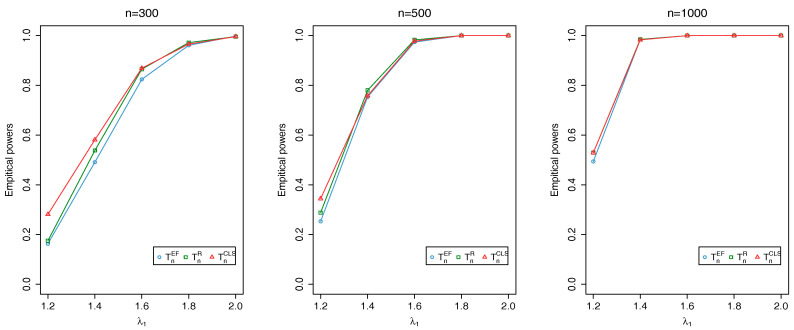
Plots of empirical powers of $T_{n}^{EF}$, $T_{n}^{R}$ and $T_{n}^{CLS}$ at nominal level 0.05 when $\lambda^{0}=1$ changes to $\lambda^{1}$ and b=4 does not change.

In order to calculate empirical sizes, observations are generated from the model (5) with

(b,λ)=(1,1),(2,1),(4,1),(8,1),(16,1)

for fixed a=4. Since the ϕ varies with *a* and *b*, we consider the various combinations of (b,λ) to detect the change of the parameters ϕ and λ. Note that ϕ tends to 1 as *b* gets close to 1. The empirical sizes are dotted in Figure 1, where the horizontal dashed lines represent the nominal level 0.05. We can conclude that the empirical sizes are adequate if the empirical sizes are located near the horizontal dashed lines. From the figure, it can be seen that none of $T_{n}^{EF}$ and $T_{n}^{R}$ has severe size distortions even for the case that *b* is close to 1. In contrast, as seen in Kang and Lee [9], $T_{n}^{CLS}$ shows sever size distortions when *b* is close to 1. Although not reported here, we could see that the results for other λ are similar to the case of λ=1. Hence, our tests remedy this defect of existing test $T_{n}^{CLS}$.

In order to examine the empirical powers, we consider the following alternatives,

$$H_{1}:\theta \;\mathrm{changes}\mathrm{from}\;\theta^{0}={\left(\phi^{0},\lambda^{0}\right)}^{T}\;\mathrm{to}\;\theta^{1}={\left(\phi^{1},\lambda^{1}\right)}^{T}\;\mathrm{at}\;t=\left[n/2\right].$$

In particular, we consider the two cases:

(i) $\lambda^{0}$ = 1 changes to $\lambda^{1}$ = 1.2, 1.4, 1.6, 1.8, 2.0 and *b* = 4 dose not change.
(ii) $b^{0}$ = 8 changes to $b^{1}$ = 7, 6 and λ changes in the same way as in (i).

**Figure 2:**
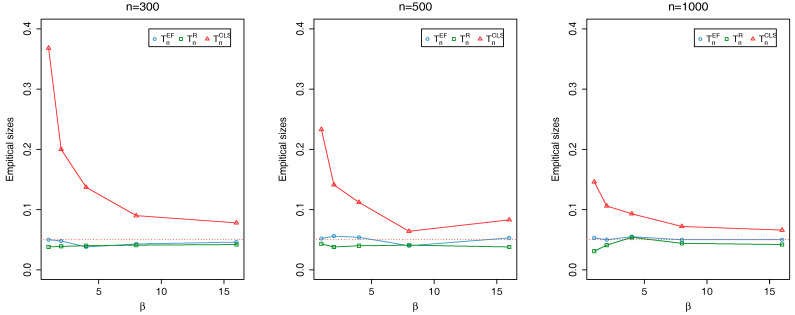
Plots of empirical sizes of $T_{n}^{EF}$, $T_{n}^{R}$ and $T_{n}^{CLS}$ at nominal level 0.05.

**Figure 3:**
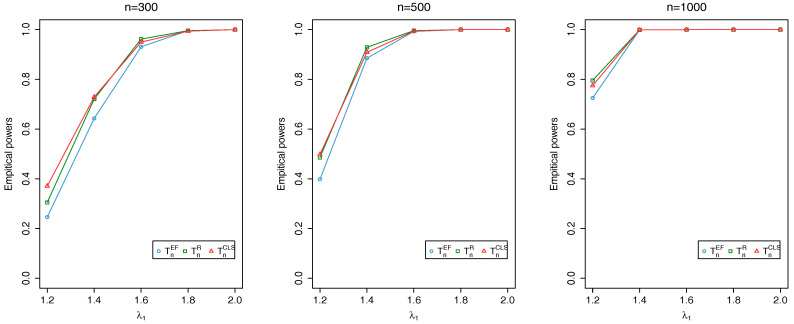
Plots of empirical powers of $T_{n}^{EF}$, $T_{n}^{R}$ and $T_{n}^{CLS}$ at nominal level 0.05 when $\lambda^{0}=1$ changes to $\lambda^{1}$ and $b^{0}=8$ changes to $b^{1}=7$.

**Figure 4:**
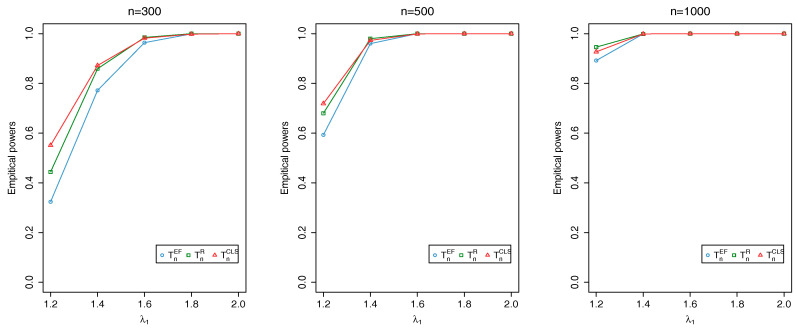
Plots of empirical powers of $T_{n}^{EF}$, $T_{n}^{R}$ and $T_{n}^{CLS}$ at nominal level 0.05 when $\lambda^{0}=1$ changes to $\lambda^{1}$ and $b^{0}=8$ changes to $b^{1}=6$.

Figure 2, Figure 3 and Figure 4 show that all the tests produce reasonably good powers and the power increases as either the distance between $\theta^{0}$ and $\theta^{1}$ or *n* increases. Overall, our simulation results demonstrate the validity of $T_{n}^{EF}$ and $T_{n}^{R}$.

## 5. Real Data Analysis

**Figure 5:**
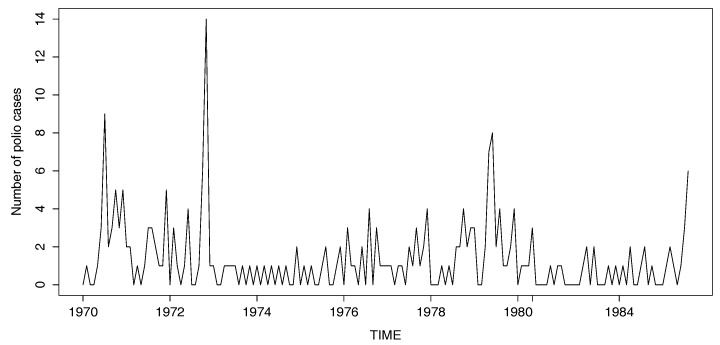
Plot of the number of polio cases in US from January 1970 to December 1983.

In this section, we apply the proposed tests in Section 3 to analyze the monthly counts of poliomyelitis cases in the US from January 1970 through December 1983, as reported by the Centers for Disease Control and Prevention. The polio incidence data is one of the most famous data sets in the context of time series of counts. This data set has been previously studied by many researchers, such as Zeger [29], Davis et al. [30], Jung and Tremayne [31] and Kang and Lee [5,9]. The data are plotted in Figure 5 and consist of 168 observations. By investigating the sample ACF and by observing the spikes, we fit the RCINAR model to the polio incidence data and examine whether a parameter change exists or not.

**Figure 6:**
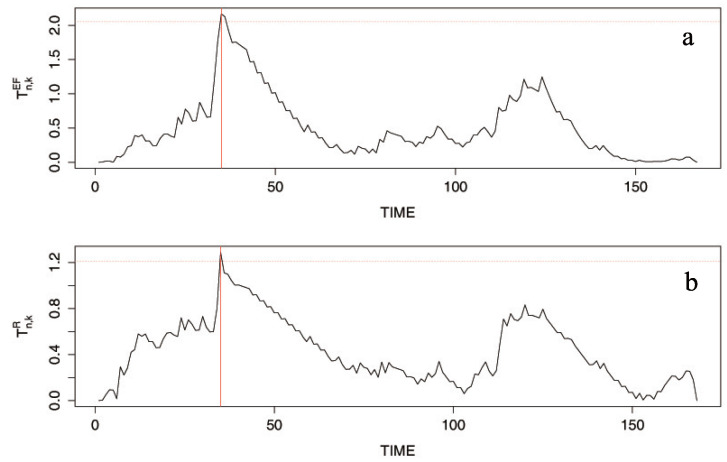
Plots of $T_{n,k}^{EF}$ (**a**) and $T_{n,k}^{R}$ (**b**) with change at $\hat{k}=35$.

In order to test for a change in (ϕ,λ), $T_{n}^{EF}$ and $T_{n}^{R}$ are performed at the nominal level 0.1; the corresponding critical values are 2.054 and 1.212, respectively (cf. the horizontal lines in Figure 6). As a result, we obtain $T_{168}^{EF}$ = 2.166 and $T_{168}^{R}=1.29$ indicating rejection of the null hypothesis. Since both $T_{168,k}^{EF}$ and $T_{168,k}^{R}$ have a maximum at k=35 (cf. Figure 6), the location of the change can be estimated as November 1972. It is the same result as those of Kang and Lee [5,9].

As we have already seen in Kang and Lee [9], it is revealed that the data in the first period, from January 1970 through October 1972, follows RCINAR model with

$$\begin{array}{c} \hat{\phi }=0.1551\left(0.0570\right),\;\hat{\lambda }=1.7949\left(0.1713\right), \end{array}$$

whereas the data in the second period follows RCINAR model with

$$\begin{array}{c} \hat{\phi }=0.1760\left(0.1029\right),\;\hat{\lambda }=0.8692\left(0.1108\right). \end{array}$$

Meanwhile, if the change is ignored and the RCINAR(1) model is fitted to the whole observations,

$$\begin{array}{c} \hat{\phi }=0.3021\left(0.1378\right),\;\hat{\lambda }=0.9511\left(0.1462\right). \end{array}$$

The figures within the parentheses denote the corresponding standard errors.

**Figure 7:**
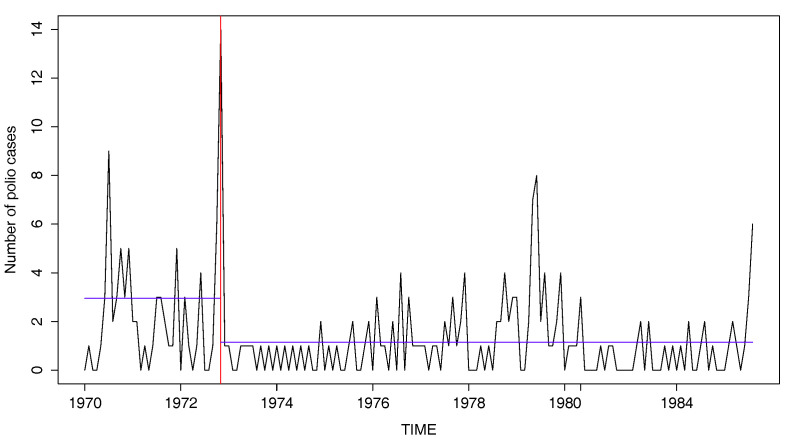
Plot of the number of polio cases with change in November 1972.

It can be seen that the estimated parameters in the first period are different from those in the second period. This indicates that ignoring a parameter change can lead to a false result. Furthermore, Figure 7 displays the polio series with the horizontal lines indicating the sample means of the first and second periods, which are 2.95 and 1.15, respectively. It looks quite evident that the series before and after November 1972 have different levels. Overall, the existence of a change is supported in this data.

## 6. Conclusions

In this study, we constructed an estimating function-based test and residual-based CUSUM test to detect a parameter change in RCINAR models and derived their limiting null distributions. According to simulation results, the proposed tests produce stable sizes even for the case that true parameter lies near the boundary of parameter space and reasonably good powers. Additionally, through a real data analysis, we demonstrated that there exists a parameter change in polio incidence data, which is consistent with previous research. Therefore, our tests can be useful tools in detecting for parameter change.

We anticipate that our tests can extend to other types of integer-valued models. Although we only derived asymptotic null distributions of the proposed tests, the behavior under the alternative, i.e., the consistency of the tests, is also of great interest. Indeed, there are several studies such as Pap and Szabó [10], Hudecová et al. [15] and Doukhan and Kengne [11] dealing with the consistency of each test in time series of counts. As with their studies, we presume that our tests also have the consistency property based on our simulation results (not reported). We leave these issues as a task for our future study.

## Appendix A

In this appendix, we provide proofs for the Theorems 2 and 3 in Section 3.

**Lemma** **A1.** *Under* $H_{0}$,

$$\begin{array}{c} \frac{1}{2\sqrt{n}}\partial_{\theta }S_{\left[ns\right]}\left(\theta_{0}\right)-\frac{\left[ns\right]}{n}\frac{1}{2\sqrt{n}}\;\partial_{\theta }S_{n}\left(\theta_{0}\right)\overset{w}{⟶}W^{\frac{1}{2}}B_{2}^{\circ }\left(s\right)\;\mathrm{in}\;\;D\;\left(\left[0,1\right],\;R^{2}\right). \end{array}$$

**Proof** **of** **Lemma** **A1.** Note that $E\left(\partial_{\theta }\epsilon_{t}^{2}\left(\theta_{0}\right)|F_{t-1}\right)=0$. Since $\left\{\partial_{\theta }\epsilon_{t}^{2}\left(\theta_{0}\right)\right\}$ is strictly stationary and ergodic, it follows from the functional limit theorem for martingales that

$$\frac{1}{\sqrt{n}}\sum_{t=1}^{\left[ns\right]}\partial_{\theta }\epsilon_{t}^{2}\left(\theta_{0}\right)\overset{w}{⟶}{\left[E\left(\partial_{\theta }\epsilon_{t}^{2}\left(\theta_{0}\right)\partial_{\theta^{T}}\epsilon_{t}^{2}\left(\theta_{0}\right)\right)\right]}^{\frac{1}{2}}B_{2}\left(s\right)\;\mathrm{in}\;\;D\;\left(\left[0,1\right],\;R^{2}\right),\;$$

that is,

$$\frac{1}{2\sqrt{n}}\partial_{\theta }S_{\left[ns\right]}\left(\theta_{0}\right)\overset{w}{⟶}W^{\frac{1}{2}}B_{2}\left(s\right)\;\mathrm{in}\;\;D\;\left(\left[0,1\right],\;R^{2}\right),$$

where $B_{2}$ is a 2-dimensional standard Brownian motion. Furthermore, by the martingale central limit theorem, we have

(A1) $$\begin{array}{c} \frac{1}{2\sqrt{n}}\;\partial_{\theta }S_{n}\left(\theta_{0}\right)\overset{d}{⟶}N\left(0,W\right). \end{array}$$

This completes the proof. ☐

**Lemma** **A2.** *Under* $H_{0}$,

$$\begin{array}{c} \underset{0\leq s\leq 1}{\sup}∥\frac{1}{2n}\partial_{\theta }^{2}S_{\left[ns\right]}\left(\theta_{0}\right)V^{-1}\;\frac{1}{2\sqrt{n}}\;\partial_{\theta }S_{n}\left(\theta_{0}\right)-\frac{\left[ns\right]}{n}\frac{1}{2\sqrt{n}}\;\partial_{\theta }S_{n}\left(\theta_{0}\right)∥=o_{P}\left(1\right). \end{array}$$

**Proof** **of** **Lemma** **A2.** Note that

$$\begin{array}{c} \underset{0\leq s\leq 1}{\sup}∥\frac{1}{2n}\partial_{\theta }^{2}S_{\left[ns\right]}\left(\theta_{0}\right)V^{-1}\;\frac{1}{2\sqrt{n}}\;\partial_{\theta }S_{n}\left(\theta_{0}\right)-\frac{\left[ns\right]}{n}\frac{1}{2\sqrt{n}}\;\partial_{\theta }S_{n}\left(\theta_{0}\right)∥\\ \leq ∥V^{-1}\frac{1}{2\sqrt{n}}\;\partial_{\theta }S_{n}\left(\theta_{0}\right)∥\;\max_{1\leq k\leq n}\frac{k}{n}∥\frac{1}{2k}\partial_{\theta }^{2}S_{k}\left(\theta_{0}\right)-V∥. \end{array}$$

Since $∥\frac{1}{2}V_{n}-V∥\to 0\;a.s.$, it can be shown that

$$\begin{array}{c} \max_{1\leq k\leq \sqrt{n}}\frac{k}{n}∥\frac{1}{2k}\partial_{\theta }^{2}S_{k}\left(\theta_{0}\right)-V∥\leq \frac{1}{\sqrt{n}}\underset{n}{\sup}∥\frac{1}{2}V_{n}-V∥=o\left(1\right)\;a.s. \end{array}$$

and

$$\begin{array}{c} \max_{\sqrt{n}<k\leq n}∥\frac{1}{2k}\partial_{\theta }^{2}S_{k}\left(\theta_{0}\right)-V∥=o\left(1\right)\;a.s., \end{array}$$

which subsequently yield that

(A2) $$\begin{array}{c} \max_{1\leq k\leq n}\frac{k}{n}∥\frac{1}{2k}\partial_{\theta }^{2}S_{k}\left(\theta_{0}\right)-V∥=o\left(1\right)\;a.s.. \end{array}$$

Owing to this and (A1), the lemma is established. ☐

**Lemma** **A3.** *Under* $H_{0}$,

$$\begin{array}{c} \underset{0\leq s\leq 1}{\sup}∥\frac{1}{2n}\partial_{\theta }^{2}S_{\left[ns\right]}\left(\theta_{0}\right)V^{-1}\left(V-\frac{1}{2}V_{n}\right)\sqrt{n}\left({\hat{\theta }}_{n}-\theta_{0}\right)∥=o_{P}\left(1\right). \end{array}$$

**Proof** **of** **Lemma** **A3.** Due to (A2), we have

$$\underset{0\leq s\leq 1}{\sup}∥\frac{1}{2n}\partial_{\theta }^{2}S_{\left[ns\right]}\left(\theta_{0}\right)∥\leq \max_{1\leq k\leq n}\frac{k}{n}∥\frac{1}{2k}\partial_{\theta }^{2}S_{k}\left(\theta_{0}\right)-V∥+∥V∥=O_{P}\left(1\right),$$

together with the facts that $∥\frac{1}{2}V_{n}-V∥\to 0\;a.s.$ and $\sqrt{n}\left({\hat{\theta }}_{n}-\theta_{0}\right)=O_{P}\left(1\right)$, the lemma is established. ☐

**Lemma** **A4.** *Under* $H_{0}$,

$$\begin{array}{c} \max_{1\leq k\leq n}\frac{1}{\sqrt{n}}\left|\sum_{t=1}^{k}\left(\epsilon_{t}\left({\hat{\theta }}_{n}\right)-\epsilon_{t}\left(\theta_{0}\right)\right)-\frac{k}{n}\sum_{t=1}^{n}\left(\epsilon_{t}\left({\hat{\theta }}_{n}\right)-\epsilon_{t}\left(\theta_{0}\right)\right)\right|=o_{P}\left(1\right). \end{array}$$

**Proof** **of** **Lemma** **A4.** Note that

$$\begin{array}{cc} & \max_{1\leq k\leq n}\frac{1}{\sqrt{n}}\left|\sum_{t=1}^{k}\left(\epsilon_{t}\left({\hat{\theta }}_{n}\right)-\epsilon_{t}\left(\theta_{0}\right)\right)-\frac{k}{n}\sum_{t=1}^{n}\left(\epsilon_{t}\left({\hat{\theta }}_{n}\right)-\epsilon_{t}\left(\theta_{0}\right)\right)\right|\\ = & \max_{1\leq k\leq n}\frac{1}{\sqrt{n}}\left|\sum_{t=1}^{k}{\left({\hat{\theta }}_{n}-\theta_{0}\right)}^{T}\partial_{\theta }\epsilon_{t}\left(\theta_{0}\right)-\frac{k}{n}\sum_{t=1}^{n}{\left({\hat{\theta }}_{n}-\theta_{0}\right)}^{T}\partial_{\theta }\epsilon_{t}\left(\theta_{0}\right)\right|\\ \leq & \sqrt{n}\left|\right|{\hat{\theta }}_{n}-\theta_{0}\left|\right|\max_{1\leq k\leq n}\frac{k}{n}∥\frac{1}{k}\sum_{t=1}^{k}\partial_{\theta }\epsilon_{t}\left(\theta_{0}\right)-\frac{1}{n}\sum_{t=1}^{n}\partial_{\theta }\epsilon_{t}\left(\theta_{0}\right)∥. \end{array}$$

Since $\partial_{\theta }\epsilon_{t}\left(\theta_{0}\right)$ is ergodic and $\sqrt{n}\left|\right|{\hat{\theta }}_{n}-\theta_{0}\left|\right|=O_{P}\left(1\right)$, the right-hand side of the inequality is $o_{P}\left(1\right)$. This completes the proof. ☐

**Lemma** **A5.** *Under* $H_{0}$,

$$\begin{array}{c} {\hat{\tau }}_{n}^{2}\overset{p}{⟶}\tau^{2}. \end{array}$$

**Proof** **of** **Lemma** **A5.** Note that

(A3) $$\begin{array}{lll} |{\hat{\tau }}_{n}^{2}-\tau^{2}| & = & \left|\frac{1}{n}\sum_{t=1}^{n}\epsilon_{t}^{2}\left({\hat{\theta }}_{n}\right)-E\left(\epsilon_{t}^{2}\left(\theta_{0}\right)\right)\right|\\ & \leq & |\frac{1}{n}\sum_{t=1}^{n}\epsilon_{t}^{2}\left({\hat{\theta }}_{n}\right)-\frac{1}{n}\sum_{t=1}^{n}\epsilon_{t}^{2}\left(\theta_{0}\right)|+|\frac{1}{n}\sum_{t=1}^{n}\epsilon_{t}^{2}\left(\theta_{0}\right)-E\left(\epsilon_{t}^{2}\left(\theta_{0}\right)\right)|. \end{array}$$

To deal with the first term of (A3), we note that

$$\begin{array}{ccc} \left|\sqrt{\frac{1}{n}\sum_{t=1}^{n}\epsilon_{t}^{2}\left({\hat{\theta }}_{n}\right)}-\sqrt{\frac{1}{n}\sum_{t=1}^{n}\epsilon_{t}^{2}\left(\theta_{0}\right)}\right| & \leq & \sqrt{\frac{1}{n}\sum_{t=1}^{n}{\left(\epsilon_{t}\left({\hat{\theta }}_{n}\right)-\epsilon_{t}\left(\theta_{0}\right)\right)}^{2}}\\ & \leq & \sqrt{\frac{1}{n}\left|\right|{\hat{\theta }}_{n}-\theta_{0}{\left|\right|}^{2}\sum_{t=1}^{n}\left|\right|\partial_{\theta }\epsilon_{t}\left(\theta_{0}\right){\left|\right|}^{2}}. \end{array}$$

Owing to $E\left({\left|\left|\partial_{\theta }\epsilon_{t}\left(\theta_{0}\right)\right|\right|}^{2}\right)<\infty$ and $\sqrt{n}\left|\right|{\hat{\theta }}_{n}-\theta_{0}\left|\right|=O_{P}\left(1\right)$, the right hand side of the above last inequality is $O_{P}\left(1/\sqrt{n}\right)$. Furthermore, it follows from the ergodicity of $\epsilon_{t}^{2}\left(\theta_{0}\right)$ that the second term of (A3) is $o_{P}\left(1\right)$, and therefore, the lemma is validated. ☐

## References

[1] Fokianos K. (2011). Some recent progress in count time series. Statistics.

[2] McKenzie E., Shanbhag D.N., Rao C.R. (2003). Discrete variate time series. Stochastic processes: Modelling and simulation. Handbook of Statistics.

[3] Weiß C.H. (2008). Thinning operations for modeling time series of counts a survey. AStA Adv. Stat. Anal..

[4] Scotto M.G., Weiß C.H., Gouveia S. (2015). Thinning-based models in the analysis of integer-valued time series: A review. Stat. Model..

[5] Kang J., Lee S. (2014). Parameter change test for Poisson autoregressive models. Scand. J. Stat..

[6] Fokianos K., Fried R. (2010). Interventions in INGARCH processes. J. Time Ser. Anal..

[7] Fokianos K., Fried R. (2012). Interventions in log-linear Poisson Autoregression. Stat. Model..

[8] Szabó T.T. (2011). Test statistics for parameter changes in INAR(*p*) models and a simulation study. Aust. J. Stat..

[9] Kang J., Lee S. (2009). Parameter change test for random coefficient integer-valued autoregressive processes with application to polio data analysis. J. Time Ser. Anal..

[10] Pap G., Szabó T.T. (2013). Change detection in INAR(*p*) processes against various alternative hypotheses. Commun. Stat. Theory Methods.

[11] Doukhan P., Kengne W. (2015). Inference and testing for structural change in general Poisson autoregressive models. Electron. J. Stat..

[12] Hudecová Š., Hušková M., Meintanis S.G., Steland A., Rafajlowicz E., Szajowski K. (2015). Detection of changes in INAR models. Stochastic Models, Statistics and Their Applications.

[13] Hudecová Š., Hušková M., Meintanis S.G. (2015). Tests for time series of counts based on the probability generating function. Statistics.

[14] Hudecová Š., Hušková M., Meintanis S.G., Cao R., Gonzalez Manteiga W., Romo J. (2016). Change detection in INARCH time series of counts. Nonparametric Statistics.

[15] Hudecová Š., Hušková M., Meintanis S.G. (2017). Tests for structural changes in time series of counts. Scand. J. Stat..

[16] Kang J., Song J. (2017). Score test for parameter change in Poisson autoregressive models. Econ. Lett..

[17] Zheng H.T., Basawa I.V., Datta S. (2007). The first order random coefficient integer-valued autoregressive processes. J. Stat. Plan. Inference.

[18] Leonenko N.N., Savani V., Zhigljavsky A.A. (2007). Autoregressive negative binomial processes. Ann. ISUP.

[19] Gomes D., e Castro L.C. (2009). Generalized integer-valued random coefficient for a first order structure autoregressive (RCINAR) process. J. Stat. Plan. Inference.

[20] Negri I., Nishiyama Y. (2017). Z-process method for change point problems with applications to discretely observed diffusion processes. Stat. Methods Appl..

[21] Horváth L., Parzen E. (1994). Limit theorems for Fisher-score change processes. Lect. Notes Monogr. Ser..

[22] Berkes I., Horváth L., Kokoszka P. (2004). Testing for parameter constancy in GARCH(p,q) models. Stat. Probab. Lett..

[23] Song J., Kang J. (2018). Parameter change tests for ARMA-GARCH models. Comput. Stat. Data Anal..

[24] Lee S., Tokutsu Y., Maekawa K. (2004). The cusum test for parameter change in regression models with ARCH errors. J. Jpn. Stat. Soc..

[25] Kulperger R., Yu H. (2005). High moment partial sum processes of residuals in GARCH models and their applications. Ann. Stat..

[26] Steutal F., Van Harn K. (1979). Discrete analogues of self decomposability and stability. Ann. Probab..

[27] Klimko L.A., Nelson P.I. (1978). On conditional least squares estimation for stochastic processes. Ann. Stat..

[28] Freeland R.K., McCabe B.P. (2004). Analysis of low count time series data by Poisson autoregression. J. Time Ser. Anal..

[29] Zeger S.L. (1988). A regression model for time series of counts. Biometrika.

[30] Davis R.A., Dunsmuir W., Wang Y. (2000). On autocorrelation in a Poisson regression model. Biometrika.

[31] Jung R.C., Tremayne A.R. (2011). Useful models for time series of counts or simply wrong ones?. AStA Adv. Stat. Anal..

